# Lactoferrin Directly Scavenges Hydroxyl Radicals and Undergoes Oxidative Self-Degradation: A Possible Role in Protection against Oxidative DNA Damage

**DOI:** 10.3390/ijms15011003

**Published:** 2014-01-14

**Authors:** Yuki Ogasawara, Megumi Imase, Hirotsugu Oda, Hiroyuki Wakabayashi, Kazuyuki Ishii

**Affiliations:** 1 Department of Analytical Biochemistry, Meiji Pharmaceutical University, 2-522-1 Noshio, Kiyose, Tokyo 204-8588, Japan; 2 Department of Hygienic Chemistry, Meiji Pharmaceutical University, 2-522-1 Noshio, Kiyose, Tokyo 204-8588, Japan; E-Mails: 7tp-al.bl_u.u_lilb.bear@ezweb.ne.jp (M.I.); ishii@my-pharm.ac.jp (K.I.); 3 Food Science & Technology Institute, Morinaga Milk Industry Co., Ltd., 5-1-83 Higashihara, Zama, Kanagawa 252-8583, Japan; E-Mails: h-oda@morinagamilk.co.jp (H.O.); h_wakaby@morinagamilk.co.jp (H.W.)

**Keywords:** lactoferrin, bovine milk, DNA damage, hydroxyl radical, UV irradiation

## Abstract

In this study, we examined the protective effect of lactoferrin against DNA damage induced by various hydroxyl radical generation systems. Lactoferrin (LF) was examined with regard to its potential role as a scavenger against radical oxygen species using bovine milk LF. Native LF, iron-saturated LF (holo-LF), and apolactoferrin (apo-LF) effectively suppressed strand breaks in plasmid DNA due to hydroxyl radicals produced by the Fenton reaction. In addition, both native LF and holo-LF clearly protected calf thymus DNA from fragmentation due to ultraviolet irradiation in the presence of H_2_O_2_. We also demonstrated a protective effect of all three LF molecules against 8-hydroxydeoxyguanosine (8-OHdG) formation in calf thymus DNA following ultraviolet (UV) irradiation with H_2_O_2_. Our results clearly indicate that native LF has reactive oxygen species-scavenging ability, independent of its nature as a masking component for transient metals. We also demonstrated that the protective effect of LF against oxidative DNA damage is due to degradation of LF itself, which is more susceptible to degradation than other bovine milk proteins.

## 1. Introduction

Lactoferrin (LF) is an 80-kDa non-heme iron-binding glycoprotein that belongs to the transferrin family [1]. In mammals, it is found at most mucosal sites and within the secondary granules of neutrophils [2–4]. Lactoferrin plays a key role in a number of the host’s first line defense mechanisms and contributes to a variety of physiological responses at both the cellular and organ level [4,5].

Lactoferrin plays a key role in immune homeostasis and functions to reduce oxidative stress at the molecular level, thus, controlling excessive inflammatory responses [6–8]. Oxidative stress occurs when the production of potentially destructive reactive oxygen species (ROS) exceeds the body’s own natural antioxidant defense mechanisms, which results in cellular damage. A cell is able to overcome and repair small perturbations; however, severe oxidative stress can lead to cell death. While moderate levels of oxidative stress can trigger apoptosis, more intense stress can lead to tissue necrosis [9–11].

Transitional metals may be mediator in the cellular response to oxidative stress. In particular, trace iron can have detrimental effects in the setting of oxidative injury. Iron crucially modulates the production of ROS by catalyzing a two-step process known as the Haber-Weiss reaction [9]. Under normal physiological conditions, the production and neutralization of ROS largely depends on the efficiency of several key enzymes, including superoxide dismutase, catalase, and glutathione peroxidase. Inefficiency of these enzymes results in overproduction of hydroxyl radicals (•OH) via the iron-dependent Haber-Weiss reaction, with a subsequent increase in lipid peroxidation. It is generally hypothesized that endogenous LF can protect against lipid peroxidation via iron sequestration. This may have significant systemic implications, as the products of lipid peroxidation, namely, hydroxyalkenals, can randomly inactivate or modify functional proteins, thereby influencing vital metabolic pathways.

Cells exposed to UV irradiation show excessive levels of ROS and DNA damage [11]. ROS-mediated oxidative damage causes DNA modification, lipid peroxidation, and the secretion of inflammatory cytokines [12]. Within DNA, 2′-deoxyguanosine is easily oxidized by ROS to form 8-hydroxy-2′-deoxyguanosine (8-OHdG) [13]. 8-OHdG is a substrate for several DNA-based excision repair systems and is released from cells after DNA repair. Thus, 8-OHdG is used extensively as a biomarker for oxidative DNA damage [14].

In the present study, we examined the protective role of LF on DNA damage caused by ROS i*n vitro*. To assess the effects of lactoferrin on various mechanisms of oxidative DNA damage, we used a UV-H_2_O_2_ system and the Fenton reaction. Our results demonstrate for the first time that LF has direct •OH scavenging ability, which is independent of its iron binding capacity and achieved through oxidative self-degradation resulted in DNA protection during •OH exposure *in vitro*.

## 2. Results

**Figure 1.**
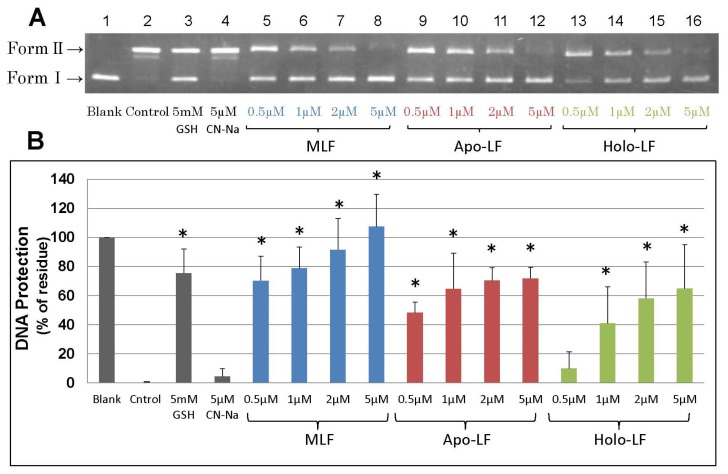
Dose response and efficacy of LFs on DNA damage by •OH generated by the Fenton reaction. Electrophoresis of plasmid DNA using an agarose gel (1.0%) was performed after exposure to •OH generated by the Fenton reaction. Experiments were conducted for 20 min at 37 °C, using iron and H_2_O_2_ (using final concentrations of 50 μL PBS, 50 μM H_2_O_2_, 5 μM FeCl_3_, 25 μM EDTA, and 10 μM ascorbic acid). (**A**) Lane 1, plasmid (Blank); lane 2, Fenton reaction mixture plus plasmid (Control); lane 3, Fenton reaction mixture plus plasmid and 5 mM GSH; lane 4, Fenton reaction mixture plus plasmid and 5 μM Casein sodium (CN-Na); lane 5, Fenton reaction mixture plus plasmid and 0.5 μM MLF; lane 6, Fenton reaction mixture plus plasmid and 1 μM MLF; lane 7, Fenton reaction mixture plus plasmid and 2 μM MLF; lane 8, Fenton reaction mixture plus plasmid and 5 μM MLF; lane 9, Fenton reaction mixture plus plasmid and 0.5 μM apo-LF; lane 10, Fenton reaction mixture plus plasmid and 1 μM apo-LF; lane 11, Fenton reaction mixture plus plasmid and 2 μM apo-LF; lane 12, Fenton reaction mixture plus plasmid and 5 μM apo-LF; lane 13, Fenton reaction mixture plus plasmid and 0.5 μM holo-LF; lane 14, Fenton reaction mixture plus plasmid and 1 μM holo-LF; lane 15, Fenton reaction mixture plus plasmid and 2 μM holo-LF; and lane 16, Fenton reaction mixture plus plasmid and 5 μM holo-LF; (**B**) DNA protection (%) was calculated based on the densitometry of EtBr-stained bands (Form I) against blank (non-treated plasmid DNA, lane 1) band intensities under the reaction conditions described in **A** (lanes 2–16). Data are presented as the mean ± S.D. of triplicate determinations. * *p* < 0.05 compared to the control value was considered as a statistically significant difference.

**Figure 2.**
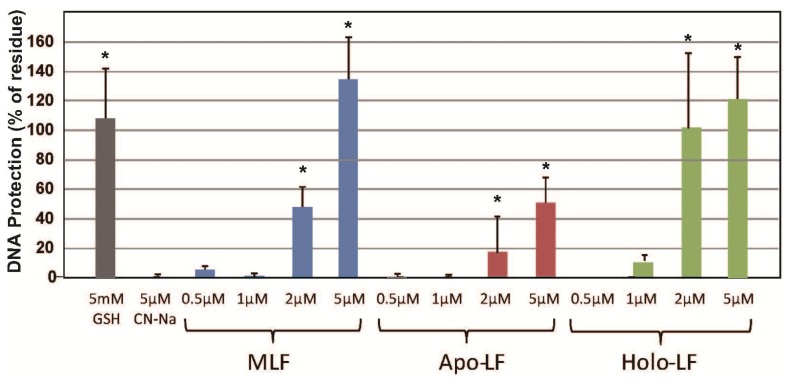
Dose responses and efficacy of LFs on calf thymus DNA strand breaks by UV irradiation in the presence of H_2_O_2_. Electrophoresis of calf thymus DNA using an agarose gel (1.0%) was performed following exposure to UV (254 nm) irradiation with 5 mM H_2_O_2_. Reactions were conducted for 10 min at room temperature. DNA protection (%) was calculated based on the densitometry of EtBr-stained bands *vs.* a non-treated sample (Control). Data are presented as the mean ± S.D. of triplicate determinations. * *p* < 0.05 compared to the CN-Na (negative control) value was considered as a statistically significant difference.

**Figure 3.**
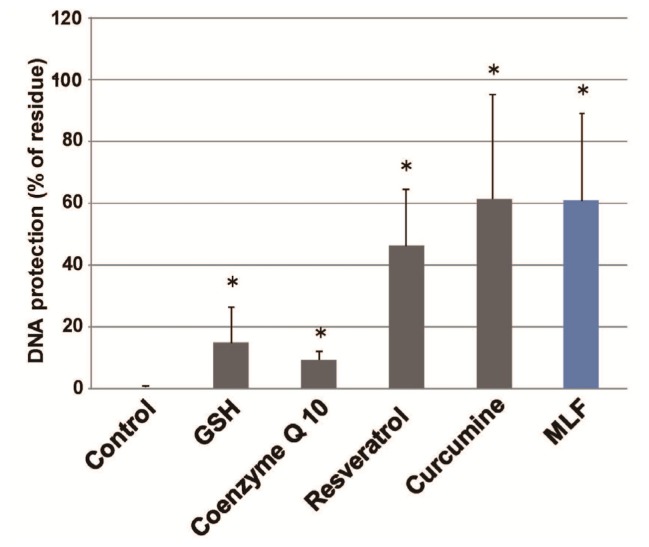
Protective effects of LFs and various antioxidants on calf thymus DNA strand breaks of p following exposure to •OH generated by the UV-H_2_O_2_ system. The effects of 5 μM MLF and various other compounds (5 mM GSH, 50 μM resveratorol, 50 μM curcumine, and 50 μM Coenzyme Q10) were determined by electrophoresis of DNA. Electrophoresis of calf thymus DNA using agarose gel (1.0%) was performed following exposure to UV irradiation (254 nm) with 5 mM H_2_O_2_ in the presence of various test compounds. Reactions were conducted for 10 min at room temperature. DNA protection (%) was calculated based on the densitometry of EtBr-stained bands *vs.* control band intensities. Data are presented as the mean ± S.D. of triplicate determinations. * *p <* 0.05 compared to the control value was considered as a statistically significant difference.

As shown in Figure 1A, the protective effect of native LF against strand breaks of plasmid DNA by the Fenton reaction showed dose-dependent behavior. Both, apo-LF and holo-LF, exerted clear protective effects; however, these were significantly less than the protection provided by native LF at low concentrations (0.5 μM). Moreover, the DNA-protective effects of LFs were equivalent to or greater than the protective effect of 5 mM GSH at a concentration of 1–5 μM (Figure 1B). To determine whether the masking ability of LF for transient metal was essential for DNA protection, we adapted a UV-H_2_O_2_ system capable of generating hydroxyl radical independent on the presence of transient metals. Figure 2 shows the protective effects of the LFs against calf thymus DNA strand breaks of plasmid DNA following UV irradiation for 10 min. Cleavage was markedly suppressed in the presence of native LF and holo-LF. As shown in Figure 3, the ability of 5 μM LF to protect against DNA damage was equivalent to or greater than that of 5 mM GSH, 50 μM resveratrol, 50 μM curcumin, and 50 μM Coenzyme Q10, using the UV-H_2_O_2_ system.

**Figure 4.**
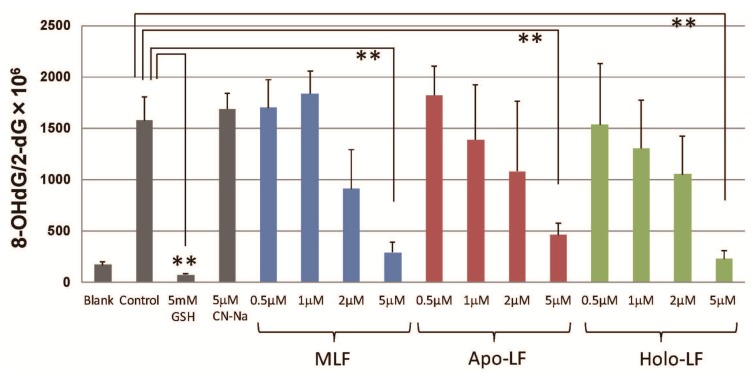
Effects of LFs on 8-OHdG formation following exposure to •OH generated by the UV-H_2_O_2_ system. 8-OHdG formation in calf thymus DNA following UV irradiation (254 nm) in the presence of H_2_O_2_ was determined as described in the Materials and Methods Section. Reactions with or without LFs were conducted for 5 min at room temperature. Data are presented as the mean ± S.D. of triplicate determinations. ** *p <* 0.01 compared to the control value obtained was considered as a statistically significant difference.

8-OHdG formation as a marker of oxidative DNA modification in calf thymus DNA was also observed following UV irradiation in the presence of H_2_O_2_. Figure 4 shows the effects of the LFs on 8-OHdG formation in calf thymus DNA, in response to hydroxyl radicals generated by the UV-H_2_O_2_ system. In comparison with control samples not containing LF, significant reductions in 8-OHdG formation were observed within calf DNA after UV-H_2_O_2_ exposure in the presence of native LF, apo-LF, and holo-LF. These results indicate that chelation of iron was not essential for the observed reduction in oxidative DNA damage induced by •OHgeneration.

**Figure 5.**
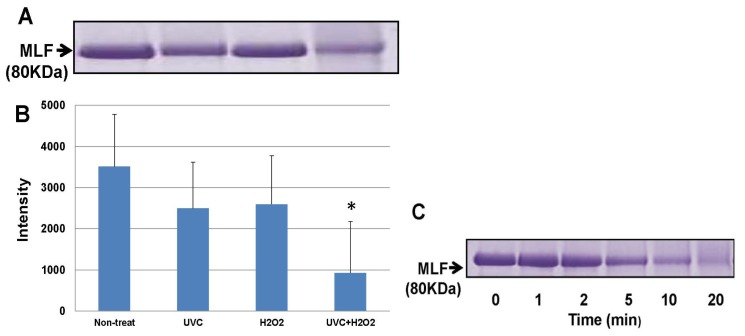
SDS gel electrophoresis of LF and apo-LF solutions exposed to UV irradiation with H_2_O_2_. (**A**) CBB stained for native LF (MLF) in SDS-polyacrylamide gel. Lane 1, non-treated; lane 2, UV (254 nm) irradiated for 10 min without H_2_O_2_; lane 3, H_2_O_2_-treated without UV irradiation; and lane 4, UV irradiated for 10 min with H_2_O_2_; (**B**) Densitometry of the stained bands demonstrated that 80-kDa native LF (MLF) remains intact under the conditions described in (**A**). Data are presented as the mean ± S.D. of triplicate determinations. * *p* < 0.05 compared to the non-treated control values obtained was considered as a statistically significant difference; (**C**) Coomassie brilliant blue (CBB) stained in SDS-polyacrylamide gel for native LF (MLF) exposed to UV (254 nm) irradiation with H_2_O_2_ for different lengths of time. Lanes from left to right: 0, 1, 2, 5, 10 and 20 min.

**Figure 6.**
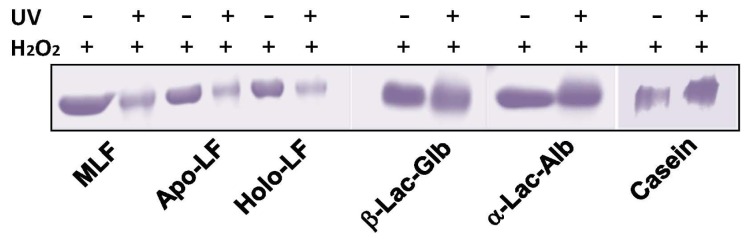
Degradation of LFs and other milk proteins exposed to UV irradiation-induced hydroxyl radicals. CBB stained for native LF (MLF), apo-LF, holo-LF, β-lactogloblin (Lac-Glb), and α-lactoalbumin (Lac-Alb), in SDS-polyacrylamide gel (5%–20%). Each protein was treated with or without UV-irradiation in the presence of 5 mM H_2_O_2_ for 10 min.

To establish the mechanism by which LF protects against DNA damage, we then examined alterations within the LF polypeptide itself during the protective reaction in the UV-H_2_O_2_ dependent •OHgeneration. As shown in Figure 5A, the LF molecules themselves were degraded or partially aggregated after exposure to UV irradiation in the presence of H_2_OWhen the samples were exposed to UV irradiation over the indicated time periods, time-dependent degradation of native LF was clearly observed (Figure 5B). Moreover, native LF was more susceptible to •OH than β-lactogloblin, α-lactoalbumin, and casein (Figure 6).

## 3. Discussion

Studies on LF, using various cancer cell lines and animal models, have recently been reviewed by Tsuda *et al.* [15]. Human clinical trials of oral LF, for the prevention of colonic polyps, have been demonstrated efficacy and have shown that dietary compounds can have direct physiological effects [16]. While a clear role of LF in cancer prevention has been demonstrated by several researchers [15,17], the potential mechanisms by which this occurs are not fully understood. Thus, there is a need to further examine the potential role of LF in moderating oxidative stress in distant organs. The aim of the present study was to clarify whether LF protects against DNA double strand breaks as a result of an iron-dependent reaction, as well as an ultraviolet irradiation-induced reaction with H_2_O_2_.

We evaluated oxidative damage to biomolecules (e.g., DNA, protein, and lipid) in the setting of •OH generated by the Fenton reaction, as well as in the setting of UV irradiation (254 nm) with H_2_O_2_. The extent of DNA damage was determined by measuring cleavage using agarose gel electrophoresis and a HPLC-ECD assay examining the formation of 8-OHdG.

Here, we report that ultraviolet irradiation with H_2_O_2_ induced the formation of 8-OHdG in calf thymus DNA. The accumulation of 8-OHdG, a hallmark of oxidative DNA damage, increased linearly up to 25 kJ/m^2^ and was dependent on the presence of oxygen within the solution. The hydroxyl radical scavenger GSH quenched the formation of 8-OHdG produced by DNA oxidation. It has been theorized that 8-OHdG formation as a result of UV irradiation proceeds via a singlet oxygen mechanism rather than by generating hydroxyl radicals [18]. The UV-H_2_O_2_ system induces 8-OHdG formation independent on the transient metals, thereby generating •OH from H_2_O_2_. The presence of lactoferrin substantially reduced 8-OHdG formation in the setting of UV irradiation and as a result of the Fenton reaction, indicating that LF has the ability to specifically quench ^1^O_2_ as well as •OH independent of its chelating ability.

We have previously demonstrated that LF inhibits the formation of a thiobarbituric acid-reactive substance in an iron/ascorbate-induced liposomal phospholipid peroxidation system, and that the inhibitory effects of LF are mediated by 9-mer peptides within the core sequence of lactoferrin, which differs from its iron binding sites [19]. Our novel findings suggest that LF might suppress oxidative DNA damage by scavenging ROS independent of its iron chelating activity. Thus, we examined whether UV irradiation-dependent generation of •OH causes susceptibility degradation or aggregation of native LF. Indeed, oxidative degradation of LF was observed using the UV-H_2_O_2_ system in the present study (Figure 5). Furthermore, degradation of all three types of LF was confirmed in this circumstance, while levels of other major milk proteins were not clearly affected by exposure to •OH using this system (Figure 6). These results suggest the possibility that LF molecules contain a specific structure that interacts with oligonucleotides to protect DNA from direct oxidative damage [20,21].

Interestingly, a recent study has demonstrated that the injection of LF before gamma-irradiation of rats reduces some cerebral symptoms of acute radiation disease [22]. It has also been shown that bLF is taken up into the nucleus via bLF receptors in human enterocyte cell lines [23]. We therefore expect that the mechanism by which LF protects against radiation exposure, including gamma irradiation, is close to being elucidated.

## 4. Experimental Section

### 4.1. Materials

Bovine LF (Fe-saturated; 17.3%) was supplied by Morinaga Co. (Kanagawa, Japan) and was stored at −20 °C. Apo-LF (Fe-saturated: 3.5%) and holo-LF (Fe-saturated: 83.6%) from bovine LF were prepared according to the method of Wakabayashi *et al.* [24]. Hydrogen peroxide solution was obtained from KANTO Chemical Co. (Tokyo, Japan). Other reagents were obtained from Sigma-Aldrich (St. Louis, MO, USA)

### 4.2. DNA Double Strand Breaks

A DNA strand cleavage assay was performed according to the method of Kukielka [25,26], with the minor modification of using pBluescript II SK^−^ DNA. Hydroxyl radicals were generated by incubating the following reagents in 0.5 mL of PBS (pH 7.4) at 37 °C for 20 min: 50 μM H_2_O_2_, 5 μM FeCl_3_, 25 μM EDTA, 10 μM ascorbic acid, and 0.5 μg of DNA. The iron salt was premixed with EDTA before addition to the reaction mixture, and the reaction was started by the addition of ascorbic acid.

### 4.3. UV Irradiation of Plasmid DNA and Calf Thymus DNA

A solution containing DNA and H_2_O_2_ was exposed to UV light for the indicated time periods to induce DNA damage. All tubes were incubated with the same amount of DNA (5 μg/mL) in the presence or absence of the test component, including LF. DNA samples were irradiated with 25 cm^2^ of UV light (254 nm) for the indicated time periods with or without native and prepared LF, apo-LF, or holo-LF. Experiments were performed at least in triplicate for all three types of LF. Ultraviolet light was generated using two 25-watt fluorescent lamps (Transilluminator Model NTFM-20; UVP, Upland, CA, USA). The tubes were mounted in a plane with their axes parallel and 4 cm apart, from which they were irradiated with UV light.

### 4.4. HPLC-EC Analysis of 8-OHdG within DNA

8-OHdG formation was determined using an HPLC-ECD system according to the method of Asami *et al.* [27]. After each exposure to UV irradiation, calf thymus DNA was isolated from the reaction mixture using a DNA-extraction kit (Wako, Osaka, Japan) according to the manufacturer’s protocol, with minor modifications to prevent the formation of 8-OHdG during DNA isolation. Isolated DNA was then digested with nucleases to obtain 8-OHdG in the nucleoside form, after which the nucleosides were injected into a Purospher^®^ STAR RP-18e (5 μm, 4.0 × 250 nm, Merck Chemicals, Darmstat, Germany) connected to an HPLC system. The latter system consisted of a HITACHI (Tokyo, Japan) L-2130 pump and a UV 7000 detector (EYELA, Tokyo, Japan). Electrochemical detection was accomplished using an ECD (Coulochem^®^ III, Guard Cell 5020; ESA Inc., Dionex, Tokyo, Japan). The mobile phase consisted of 0.2 M Na_2_PO_4_ containing 6% methanol. The flow rate was 1.0 mL/min with the following applied conditions: *E*1: 150 mV, *R*: 1 μA, Filter: 10 s, output: 1.0 V, *E*2: 300 mV, *R*: 50 μA, Filter: 10 s, and output: 1.0 V. DNA-specific 8-OHdG was expressed in terms of the ratio of 8-OHdG to deoxyguanosine (2dG).

### 4.5. Oxidative Alteration of LF by Exposure to Hydroxyl Radicals

Molecular changes to LFs, β-lactogloblin, α-lactoalbumin, and casein after exposure to hydroxyl radicals induced by the UV-H_2_O_2_ system were demonstrated by SDS-polyacrylamide gel (5%–20%) electrophoresis followed by staining with Coomassie brilliant blue (CBB). The stained gels were image scanned, after which the stained bands were analyzed using the gel image analyzer software (ATTO, Tokyo, Japan).

### 4.6. Statistical Analysis

Values are presented as the mean ± SD. The data were evaluated using the Student’s *t*–test (*p* < 0.05 was considered as a statistically significant difference).

## 5. Conclusions

In conclusion, our findings strongly indicate that LF acts, not only as a transient metal chelator, but also as a sacrificial scavenger for ROS, and that it protects through direct interaction with hydrogen radicals, resulting in degradation of LF itself. This may be enabled by the structural characteristics of LF, which has a high affinity for binding DNA. It therefore seems reasonable that endogenous production of LF might protect against •OH exposure initiated by a range of environmental factors including UV irradiation in order to protect against cellular oxidative injury.

## Abbreviations

LF: lactoferrin
EDTA: ethylenediaminetetraacetic acid
ROS: reactive oxygen species
8-OHdG: 8-hydroxydeoxyguanosine
holo-LF: iron-saturated lactoferrin
apo-LF: apolactoferrin
MLF: native milk lactoferrin
